# Predicting Tuberculosis Outcomes Using Routine Surveillance Data in Chiang Mai, Thailand: Retrospective Cohort Study

**DOI:** 10.2196/86495

**Published:** 2026-06-25

**Authors:** Porramat Saksaen, Ekkarat Boonchieng, Aksara Thongprachum, Surasak Maotheuak, Sineenart Chautrakarn, Waraporn Boonchieng

**Affiliations:** 1 Faculty of Public Health, Chiang Mai University, 239 Huay Kaew Road, Muang District, Chiang Mai, 50200, Thailand, 66 53 942525; 2 Faculty of Science, Department of Computer Science, Chiang Mai University, Chiang Mai, Thailand; 3 Faculty of Education, Department of Curriculum, Teaching & Learning, Chiang Mai University, Chiang Mai, Thailand

**Keywords:** tuberculosis, predictive modeling, treatment delay, mortality, health equity, resource-limited settings, sustainable development goals, SDG, SDG 3, SDG 10, Thailand, good health and well-being, reduced inequalities

## Abstract

**Background:**

Tuberculosis (TB) remains one of the leading causes of death from a single infectious disease worldwide. In Thailand, persistent gaps in early detection and access to TB care remain important public health challenges, particularly among populations in rural and remote areas.

**Objective:**

This study aimed to develop and evaluate predictive models for TB outcomes using routine surveillance data to support risk stratification, thereby informing public health decision-making in Chiang Mai Province, Thailand.

**Methods:**

A retrospective cohort study was conducted using data from 5557 TB cases registered in the National Tuberculosis Information Program in Chiang Mai Province from 2020 to 2024. Models were developed to predict treatment success, mortality, and time to treatment initiation. We evaluated model performance using the area under the receiver operating characteristic curve, the Harrell Concordance Index, and error-based metrics for time-to-treatment prediction. Scenario analyses were conducted under predefined assumptions to assess projected changes in detection-related indicators, treatment coverage, and mortality relative to provincial baseline indicators.

**Results:**

The models demonstrated good predictive performance. Mortality was associated with HIV co-infection (hazard ratio 5.80) and was highest among older adults with HIV co-infection (hazard ratio 12.30). Treatment delays were longer among individuals living in rural or remote areas and among those without health insurance, with mean delays ranging from 8 to 18 days. These findings suggest distinct patterns in TB outcomes, with mortality more closely related to clinical vulnerability and treatment delay more closely related to health care access factors. Under modeled scenarios, the models projected that detection-related indicators would increase by 25%, treatment coverage would increase by 15%, and mortality would decrease by 20% relative to provincial baseline conditions.

**Conclusions:**

Predictive modeling using routine TB surveillance data may support risk stratification and provide insights into treatment outcomes and delays. The findings highlight the combined roles of clinical vulnerability and health care access factors in TB outcomes and support further evaluation of data-driven approaches to inform targeted TB interventions in resource-limited settings.

## Introduction

Tuberculosis (TB) remains one of the leading causes of death from a single infectious disease worldwide. The World Health Organization (WHO) estimates that 10.7 million people developed TB globally in 2024, of whom 8.3 million were diagnosed and notified, highlighting persistent gaps in case detection and reporting. Although global health efforts have made progress in TB diagnosis and treatment, global TB incidence declined by approximately 12% between 2015 and 2024, remaining well below the End TB Strategy target of a 50% reduction by 2025 [1].

Thailand is classified as a high-burden country for TB and TB/HIV by the WHO. Thailand has an estimated 113,000 new TB cases annually, corresponding to an incidence of 157 per 100,000 population. Of these, HIV co-infection accounts for 9,400 cases, and 13,000 deaths are attributed to TB each year [2]. Reported treatment coverage is approximately 71%, indicating persistent gaps in access to diagnosis and care. Previous studies have shown that TB does not distribute evenly across Thailand but rather clusters in several areas, particularly along international border regions [3]. These geographic and health-system disparities highlight the need for analytical approaches that integrate epidemiological, clinical, and health service data to support more precise and equitable TB control.

Mathematical models, particularly compartmental models such as the SEIR (susceptible-exposed-infectious-recovered) framework, have been widely used to study TB epidemiology. Unlike the classical SIR model, the SEIR framework explicitly includes an exposed compartment representing individuals who are infected but not yet infectious, thereby accounting for the latent stage before progression to active TB disease [4,5]. However, classical SEIR-based approaches often rely on simplifying assumptions, such as homogeneous mixing and fixed transition rates between compartments. These approaches may have limited ability to capture individual-level heterogeneity in clinical risk, health care access, and treatment outcomes.

Researchers are increasingly applying machine learning (ML) methods to TB-related predictive tasks, such as incidence prediction, diagnosis, and treatment outcome prediction [6,7]. These approaches can exploit high-dimensional, heterogeneous data and demonstrate strong predictive performance for specific TB-related outcomes. ML-based approaches have also been incorporated into clinical decision support systems to assist with diagnostic and treatment decision-making [8,9]. However, most existing studies focus on isolated prediction tasks and do not explicitly link individual-level risk estimation with broader epidemiological disease-stage structures.

The strengths of SEIR-based epidemiological models and ML approaches are complementary. SEIR-based frameworks [10] provide a structured way to conceptualize disease progression at the population level, whereas ML approaches enable data-driven prediction of observable individual-level outcomes from clinical and surveillance data. However, there remains limited evidence on how routine TB surveillance data can be organized within an epidemiologically informed framework to generate practical predictions of treatment outcomes, mortality, and treatment delays. This gap is particularly important in settings where surveillance systems contain rich programmatic data, but practitioners do not routinely use them for risk stratification or decision support.

TB control in Thailand is primarily implemented through health care facility–based diagnosis, registration, and treatment reporting. In contrast, routine programmatic reporting remains centered on surveillance and treatment monitoring rather than risk-prediction tools. Ongoing programmatic work includes surveillance and focused interventions for key high-risk or vulnerable populations, such as people living with HIV, prisoners, migrants, older adults, and non-Thai populations [11,12]. Thailand has established the National Tuberculosis Information Program (NTIP) as a national online TB reporting system. However, there are gaps in translating surveillance data into analytical tools that can support decision-making at different levels of the health care system.

NTIP is Thailand’s national TB reporting system that collects data on diagnoses, treatment registrations, and outcomes from health care facilities [13]. While NTIP provides a standardized national dataset, it reflects diagnosed and registered TB cases and does not capture unobserved infection or transmission processes. These characteristics make NTIP suitable for developing predictive models focused on routinely observed clinical and programmatic outcomes. Integration of NTIP data with analytical models that combine an epidemiologically informed structure and predictive modeling may support more systematic use of surveillance data for risk stratification and policy planning.

Therefore, this study aimed to develop and evaluate a TB surveillance framework based on an SEIR-informed conceptual structure and predictive models using NTIP data from Chiang Mai Province. Specifically, the study aimed to assess whether routinely collected surveillance data could support risk prediction, risk stratification, and public health decision-making for TB outcomes in a resource-limited setting.

## Methods

### Study Design

This retrospective cohort study used routine surveillance data to develop a TB surveillance framework that combines an SEIR-informed conceptual structure with individual-level predictive models. We used the SEIR framework [10,14] to guide the conceptual organization of TB disease stages. Empirical modeling focused on clinical and programmatic outcomes recorded in NTIP, including treatment success, mortality, and time to treatment initiation.

Within this framework, NTIP provided data on diagnosed and registered TB cases, treatment outcomes, deaths, and treatment initiation dates. Registered TB cases represented the observed infectious state among patients captured by the surveillance system. We operationalized recovery-related outcomes as treatment success, and we used recorded deaths to represent mortality. NTIP did not directly capture susceptible and exposed populations; therefore, we retained them as conceptual components of the SEIR-informed framework. Table 1 summarizes the observability and operationalization of SEIR-D (susceptible-exposed-infectious-recovered-death) components in NTIP data.

The study, therefore, linked an epidemiologically informed disease-stage framework with predictive modeling of observed outcomes among patients diagnosed with and registered for TB.

**Table 1.** Summarizes the observability and operationalization of SEIR-D (susceptible-exposed-infectious-recovered-death) components in the National Tuberculosis Information Program (NTIP) data^a^.

Component	Epidemiological meaning	NTIP observability	Operationalization in this study
Susceptible	Population at risk of TB^b^ infection	Not directly observed	Conceptual component of the SEIR-informed framework
Exposed	Individuals with TB infection before registered active TB disease	Not directly observed	Conceptual component of the SEIR-informed framework
Infectious	Diagnosed active TB cases	Observed among registered cases	Registered TB cases recorded in NTIP
Recovered	Successful treatment outcome	Observed	Treatment success recorded in NTIP
Death	Death during treatment or follow-up	Observed	Death recorded in NTIP treatment outcome data
Treatment delay	Interval from diagnosis to treatment initiation	Observed	Days between diagnosis and treatment initiation

a SEIR-D components were defined within an SEIR-informed conceptual framework and operationalized according to the structure of the NTIP surveillance dataset. Susceptible and exposed populations were not directly observed in NTIP and were retained as conceptual components. Empirical analyses focused on registered TB cases, treatment success, death, and time to treatment initiation. Treatment delay was considered a programmatic interval rather than a disease-state compartment.

b TB: tuberculosis.

### Study Area and Data Sources

Data were obtained from the NTIP, including records of patients with TB registered in Chiang Mai Province between October 2020 and September 2024. The NTIP database provided individual-level data on diagnosis, treatment registration, comorbidities, and treatment outcomes.

### Inclusion and Exclusion Criteria

The study population consisted of patients registered for TB treatment in NTIP during the study period. We excluded patients with missing values for key variables required for model development, including age, sex, district of residence, treatment initiation date, or treatment outcome. These variables were required to define the analytical cohort, predictor structure, and primary outcomes. The analytical dataset comprised 5557 cases after data cleaning and eligibility screening based on variable completeness. Records were excluded only when missingness involved variables essential for defining the analytical cohort, primary outcomes, or model structure; we addressed missing values in nonessential variables separately using multiple imputation.

### Data Management and Preparation

The key variables in the analytical dataset were age, sex, district of residence, date of treatment initiation, and treatment outcome. Records with missing values in key analytical variables were excluded. Missing values were imputed for nonessential variables, such as selected comorbidities and health care system indicators, using multiple imputation by chained equations, which generated 20 imputed datasets to account for the uncertainty of the imputed values and to reduce potential bias associated with complete-case analysis [14-16]. The imputation model included observed demographic, clinical, and health system variables and assumed a missing-at-random mechanism conditional on these observed variables. This approach is consistent with established recommendations for handling incomplete observational data when missingness is assumed to be conditional on observed variables [14,16].

BMI, comorbidities, HIV status, insurance status, and other predictor variables were recorded at TB registration or initial clinical assessment and used as baseline predictors for subsequent recorded outcomes. A BMI of less than 18.5 kg/m^2^ was defined using the BMI value recorded at registration or initial assessment. We used the Tukey fences and the isolation forest algorithm [17] to identify outliers. We set the contamination parameter of the isolation forest algorithm to 0.05, and we checked model outputs for clinical plausibility. Residence categories were defined using geographic accessibility to TB services, including the distance from residence to treatment facilities. Urban residence, representing the most accessible category, was used as the reference group for residence variables. All eligible records were retained for model development and assessed using train-test splitting and internal cross-validation.

### Development of Predictive Models

We conducted predictive modeling using observable outcomes from the final analytic NTIP dataset of 5557 TB cases. We selected models according to the structure of the recorded outcomes and the statistical requirements of each prediction task. We applied logistic regression for binary treatment success [17,18], the Cox proportional hazards model for time-to-event mortality with censoring [19,20], and the log-normal accelerated failure time model for time to treatment initiation [20]. This approach allowed each model to correspond to the scale, timing, and censoring structure of each outcome under analysis. Because NTIP records diagnosed and registered TB cases rather than the full susceptible or exposed population, model construction focused on clinical and programmatic outcomes available among registered patients. The SEIR framework was used to organize disease-stage concepts, consistent with compartmental modeling approaches that distinguish susceptible, exposed, infectious, and recovered states [10,21].

A multivariable logistic regression model was used to predict treatment success as a binary outcome, selected for its interpretability and suitability for clinical decision-making [17,18]. A Cox proportional hazards model [19,20] was used to estimate the hazard of death with time-to-event data, accounting for censoring. We tested the proportional hazards assumption using Schoenfeld residuals. We fitted an accelerated failure time model with a log-normal distribution to estimate delays between diagnosis and treatment initiation; this approach estimates the effect of covariates on event time rather than on the hazard function [20].

Together, these models estimated binary outcomes, time-to-event risks, and delays within a unified analytical framework based on recorded clinical and programmatic end points, including treatment success, mortality, and diagnosis-to-treatment delay. We specified the predictive models for recorded outcomes rather than for latent or unobserved transmission states. Model performance was assessed using discrimination and calibration metrics, including the area under the receiver operating characteristic curve (AUC) for classification models [22] and concordance-based measures for survival models [23].

### Model Training and Validation

We assessed model performance using a train-test split (train: 4446/5557, 80%; test: 1111/5557, 20%) and performed internal validation using 10-fold stratified cross-validation on the training set. For classification models, the AUC was used as a performance metric [22]. For survival models, the Harrell Concordance Index was used as a performance metric [23]. We assessed prediction error for the time-to-treatment model using root mean squared error and mean absolute error. Additionally, we assessed the goodness-of-fit of the logistic regression model using the Hosmer-Lemeshow test [17]. We assessed discrimination, calibration, and prediction error across outcome types and model specifications.

### Management of Conflicting Outcomes

We analyzed mortality and treatment success as separate outcome-specific end points. We modeled mortality using time-to-event methods, whereas we modeled treatment success as a binary outcome. We applied this outcome-specific modeling strategy to support risk stratification across different stages of the TB care pathway. We interpreted estimates as predictive associations rather than causal effects.

### Analysis of Scenarios

We performed scenario analyses to assess projected changes in detection-related indicators, treatment coverage, and mortality under predefined modeled assumptions. The baseline scenario represented the observed provincial TB program conditions prior to applying the modeled changes. Baseline values included the provincial detection-related indicator, treatment coverage, and mortality rate, which were derived from Chiang Mai provincial TB program indicators. We derived the time to treatment initiation from recorded diagnosis and treatment initiation dates in NTIP and modeled it separately using a log-normal accelerated failure time model.

We represented screening as a programmatic process related to case detection rather than as a disease-state compartment. We specified detection-related changes as proportional changes relative to provincial TB program baseline indicators. We represented changes in treatment processes using predefined relative changes in treatment coverage and mortality, which were applied to provincial baseline indicators.

In the baseline scenario, the detection-related indicator was 87.08%, treatment coverage was 71.90%, and mortality was 11.90% [12]. The scenario parameters for detection-related improvement, treatment coverage improvement, and mortality reduction were predefined assumptions rather than estimates from an observed intervention. We, therefore, interpreted scenario outcomes as model-based projections rather than observed intervention effects, consistent with the use of modeling approaches to inform public health decision-making in the absence of direct population-level evidence [24].

### Statistical Analysis and Software

We conducted all analyses using R (version 4.1.2; R Foundation for Statistical Computing) and Python (version 3.11; Python Software Foundation). We defined statistical significance as *P*<.05, and we reported all estimates with 95% CI. As NTIP represents a registry of reported TB cases rather than a sampled survey dataset, we therefore did not apply survey weighting. Given the analysis’s predictive and exploratory focus, we instead emphasized effect estimates, CI, and model performance metrics rather than confirmatory hypothesis testing.

### Ethical Considerations

The research ethics committee of the Faculty of Public Health, Chiang Mai University (approval: ET030/2025) and the Chiang Mai Provincial Public Health Office approved the study protocol. We anonymized all data prior to analysis and retained no identifiable personal information. Data were stored in secure, encrypted systems in accordance with applicable data protection regulations.

## Results

### Demographic, Clinical, and Health Care Access Characteristics of the Study Participants

This study analyzed 5557 TB cases recorded in the NTIP in Chiang Mai Province between 2020 and 2024. Most participants were male (3756, 67.6% cases), and the mean age was 45.3 (SD 14.8) years. HIV co-infection was present in 556 (10.0% cases), 1000 (18.0%) cases had diabetes mellitus, and 1223 (22.0%) cases had a BMI <18.5 kg/m^2^. Health care access characteristics included absence of health insurance in 445 (8.0%) cases, rural residence in 1556 (28.0%) cases, and remote residence in 445 (8.0%) cases (Table 2).

**Table 2.** Baseline demographic, clinical, and health care access characteristics of the study population (N=5557)^a^.

Characteristic	Values
Male sex, n (%)	3756 (67.6)
Age (y), mean (SD)	45.3 (14.8)
HIV-positive, n (%)	556 (10.0)
Diabetes mellitus, n (%)	1000 (18.0)
BMI (<18.5 kg/m^2^), n (%)	1223 (22.0)
No health insurance, n (%)	445 (8.0)
Rural residence, n (%)	1556 (28.0)
Remote residence, n (%)	445 (8.0)

a Baseline demographic, clinical, and health care access characteristics of National Tuberculosis Information Program–registered patients with tuberculosis in Chiang Mai Province, Thailand, 2020‐2024. Data are presented as n (%) or mean (SD).

### Predictive Model Performance

Predictive models demonstrated good performance across recorded TB outcomes. The AUC for the treatment success model was 0.88 (95% CI 0.86‐0.90), indicating good discrimination. The mortality model showed the Harrell Concordance Index of 0.86 (95% CI 0.84‐0.88), indicating good concordance between predicted and observed survival outcomes. We summarized prediction error for time to treatment initiation using root mean squared error and mean absolute error, with values of 8.2 days and 5.7 days, respectively. The logistic regression model was adequately calibrated, with no evidence of poor fit based on the Hosmer-Lemeshow test (*P*>.05; Table 3).

**Table 3.** Performance of predictive models for tuberculosis outcomes^a^.

Outcome	Model	Performance metric
Treatment success	Logistic regression	AUC^b^=0.88 (95% CI 0.86‐0.90)
Mortality	Cox proportional hazards model	Harrell Concordance Index=0.86 (95% CI 0.84‐0.88)
Time to treatment initiation	Log-normal AFT^c^ model	RMSE^d^=8.2 days; MAE^e^=5.7 days^f^

a Performance metrics of predictive models for tuberculosis outcomes in a retrospective cohort of National Tuberculosis Information Program–registered patients in Chiang Mai Province, Thailand, 2020‐2024.

b AUC: area under the receiver operating characteristic curve.

c AFT: accelerated failure time.

d RMSE: root mean squared error.

e MAE: mean absolute error.

f RMSE and MAE represent prediction errors for the time to treatment initiation, expressed in days.

### Treatment Success as a Recovery-Related Outcome

We modeled treatment success as the recovery-related outcome recorded in NTIP. The logistic regression model for treatment success demonstrated good discrimination, with an AUC of 0.88 (95% CI 0.86‐0.90), indicating that routinely recorded clinical and programmatic variables are informative for classifying treatment success among registered patients with TB.

### Factors Associated With Mortality

HIV infection was associated with higher mortality among patients with TB (HR 5.80; 95% CI 4.20‐7.90; *P*<.001). Patients aged older than 65 years with HIV co-infection had the highest estimated mortality risk (HR 12.30; 95% CI 8.90‐16.40; *P*<.001). Higher mortality was also associated with diabetes mellitus (HR 3.10; 95% CI 2.30‐4.40; *P*<.001) and a BMI of less than 18.5 kg/m^2^ (HR 2.30; 95% CI 1.70‐3.10; *P*<.001; Table 4). We present these estimates as outcome-specific associations from the mortality model.

**Table 4.** Factors associated with mortality^a^.

Factor	HR^b^ (95% CI)		*P* value
HIV-positive	5.80 (4.20‐7.90)		<.001
Age (>65 y with HIV co-infection)	12.30 (8.90‐16.40)		<.001
Diabetes mellitus	3.10 (2.30‐4.40)		<.001
BMI (<18.5 kg/m^2^)	2.30 (1.70‐3.10)		<.001

a Factors associated with mortality among patients with tuberculosis in Chiang Mai Province, Thailand, from 2020 to 2024. Hazard ratios were estimated using Cox proportional hazards models.

b HR: hazard ratio.

### Factors Associated With Treatment Delay

Treatment delays were longer among individuals living in rural and remote areas than among those living in urban areas. Rural residence was associated with an average delay of 12 (95% CI 9‐15) days, remote residence with 18 (95% CI 14‐22) days, lack of health insurance with 8 (95% CI 6‐10) days, and age >70 years with 6 (95% CI 4‐9) days (Table 5). We present treatment-delay estimates as mean differences with 95% CI.

**Table 5.** Factors associated with treatment delays^a^.

Factor	Days, mean difference (95% CI)	
Rural residence	12 (9‐15)	
Remote residence	18 (14‐22)	
No health insurance	8 (6‐10)	
Age (>70 y)	6 (4‐9)	

a Factors associated with treatment delay among patients with tuberculosis in Chiang Mai Province, Thailand, 2020‐2024. Estimates represent differences in the expected time to treatment initiation derived from the accelerated failure time model. Urban residence, the most accessible residence category, was used as the reference group for rural and remote residence estimates.

### Simulation Results of Modeled Scenarios

We derived baseline values for scenario comparisons from Chiang Mai provincial TB program indicators. The scenario analysis did not model the redistribution of specific resources between geographic risk areas. Instead, it represented potential programmatic improvements in detection-related indicators, treatment coverage, and mortality under predefined assumptions. Under these modeled scenarios, the models projected detection-related indicators to increase by 25% (95% CI 22‐28), treatment coverage by 15% (95% CI 12‐18), and mortality to decrease by 20% (95% CI 16‐24) relative to baseline conditions (Table 6). Readers should interpret these projections as scenario-based estimates rather than as observed effects of resource reallocation or implemented program changes.

**Table 6.** Projected outcomes under modeled scenarios relative to provincial baseline indicators^a^.

Outcome	Provincial baseline (%)	Projected change (%)	95% CI (%)
Detection-related indicators	87.08	+25	22‐28
Treatment coverage	71.90	+15	12‐18
Mortality	11.90	–20	16‐24

a Projected outcomes under modeled scenarios are relative to provincial baseline indicators in Chiang Mai Province, Thailand. Estimates represent model-based projections under predefined assumptions and do not reflect observed intervention effects or implemented program changes. Baseline values were obtained from provincial tuberculosis program indicators.

## Discussion

### Principal Findings

This study examined TB outcomes using routinely collected surveillance data and applied predictive modeling to identify factors associated with mortality and treatment delay. The findings suggest distinct patterns in TB outcomes, where mortality was more strongly associated with clinical vulnerability, while treatment delay was more closely related to health care access factors. By examining these outcomes within a unified surveillance-based analytical framework, this study highlights the importance of distinguishing between clinical risk factors and health care access barriers when designing TB control strategies, as these domains reflect distinct yet interacting pathways that influence patient outcomes.

The SEIR-informed framework provided a structure for organizing disease-stage concepts, and the predictive models focused on recorded clinical and programmatic outcomes in NTIP, including treatment success, mortality, and time to treatment initiation. This approach linked an epidemiological disease-stage perspective with practical risk stratification using routinely collected surveillance data.

### Comparison With Previous Studies

The association between HIV co-infection and higher mortality observed in this study (HR=5.80) is consistent with prior survival analyses showing reduced survival among patients with TB or HIV coinfection [25] and cohort evidence identifying HIV positivity as a predictor of mortality among patients with TB [26]. The relatively high magnitude of association observed in this study may, therefore, reflect not only HIV-related clinical vulnerability and the increased complexity of clinical management in patients with coinfection, but also contextual factors such as diagnostic timing and health system access, which previous studies have identified as contributors to diagnostic delays and poorer TB outcomes [27].

In addition to HIV co-infection, baseline metabolic and nutritional factors also appear relevant to TB outcomes. The associations observed for diabetes are consistent with data from prospective cohorts, indicating that diabetes and prediabetes are associated with adverse TB treatment outcomes [28]. Moreover, prior research has linked dysglycemia to more severe disease presentation, including higher radiographic severity and increased bacillary load, as well as increased TB lethality among patients with diabetes [29]. Low BMI may indicate nutritional vulnerability and should be interpreted in the context of a broader clinical risk profile rather than as an independent predictor, consistent with survival analyses demonstrating the significance of baseline clinical characteristics in shaping TB mortality risk [26].

In addition to these clinical risk factors, health system and access-related barriers also contributed to differences in TB outcomes. Regarding treatment delay, the finding that rural and remote populations experienced longer delays is consistent with systematic review evidence indicating that delays in diagnosis and treatment of TB continue to be important barriers to TB control [27]. One suggested approach to reduce these access gaps is active case-finding, particularly through community-based approaches [30] and screening in general or at-risk populations [31]. However, these approaches are likely to be most effective when implemented with appropriate coverage and linked to timely diagnosis and treatment initiation. These findings suggest that geographic variation in treatment delay may reflect underlying disparities in health care access and care pathways rather than solely spatial disease patterns.

### Interpretation and Implications

From a methodological perspective, the results are consistent with a broader pattern in the TB modeling literature, in which different modeling approaches capture different aspects of the disease process. Researchers have applied ML methods to address several TB-related predictive tasks, including diagnostic classification with high-dimensional serological data [32], incidence prediction using meteorological and air pollution data [6], and treatment outcome prediction using routinely available clinical indicators [33]. Recent work has also examined the use of ML for TB incidence forecasting within broader epidemiological modeling frameworks [34]. Such approaches are often designed to optimize predictive accuracy; however, they may have limited interpretability regarding underlying disease mechanisms, particularly when the observed data include only diagnosed and reported cases rather than the full spectrum of transmission dynamics.

Mechanistic models, particularly SEIR-based approaches, provide a structured framework for conceptualizing disease progression and population-level disease dynamics [35] and have been extended to examine TB dynamics under different modeling assumptions [36]. Nevertheless, such models often rely on simplifying assumptions and may not fully capture real-world heterogeneity or individual-level risk. In this study, we used the SEIR framework conceptually to organize disease stages, while applying predictive models to recorded outcomes in routine surveillance data. The analysis, therefore, focused on recorded clinical and programmatic outcomes rather than population-level transmission dynamics. This approach supports alignment between theoretical disease-stage concepts and the structure of routinely available surveillance data.

Recent advances in hybrid TB modeling suggest that integrating mechanistic models with ML may provide a more balanced framework that supports predictive performance while maintaining epidemiological interpretability [37]. In this study, this integration was applied at a conceptual level, reflecting the structure and constraints of routine surveillance data. The study demonstrates a practical approach to organizing surveillance data within an epidemiologically informed framework to support the prediction of recorded TB outcomes and risk stratification.

The literature has well established that TB epidemiology exhibits spatial heterogeneity, with geographic variation observed across spatial and socioeconomic contexts [3,38]. More recently, work using satellite imagery and urban density measures has shown that spatial proxies may help identify areas for targeted active case-finding, while highlighting the need for cautious interpretation of geographic risk patterns [39]. The observed association between geographic residence and treatment delay suggests that spatial disparities in TB outcomes are attributable to differences in health care access and service availability. However, spatial clustering does not necessarily reflect transmission dynamics, and geographic variation may also be due to differences in health care access, reporting systems, population structure, and socioeconomic conditions. This distinction is important because outcomes may vary geographically due to both epidemiological and health-system factors.

From a programmatic perspective, the findings suggest that interventions targeting both early case detection and reduction of structural barriers to health care access may be needed to improve TB outcomes, rather than applying a uniform strategy across all populations. This distinction has implications for intervention design, as strategies to address clinical risk (eg, comorbidity management) may differ substantially from those intended to address access barriers (eg, service decentralization or active case-finding). This interpretation is consistent with the scenario analyses presented in this study, which show that improvements in detection and treatment processes could be associated with changes in projected outcomes under modeled assumptions. These projections provide scenario-based estimates of possible implications for programs, and readers should interpret them in the context of the assumptions and baseline indicators used in the analysis.

Targeted case-finding strategies may help improve access to diagnosis, especially in high-risk populations. However, delays in TB care are multifactorial and influenced by both patient- and health system–level factors [27,30]. The effectiveness of these approaches may, therefore, depend on both expanded access and integration into routine care pathways. In practical terms, predictive outputs may help TB programs prioritize active follow-up, outreach screening, referral support, patient navigation, and closer monitoring for clinically vulnerable patients or those living in underserved areas. Further implementation research is needed to determine whether these outputs should guide additional resources, the redistribution of existing resources, or both. Digital health technologies and clinical decision support systems may facilitate implementation [8,40], but future studies should evaluate their practical impact in real-world clinical and public health settings.

### Strengths

This study used a large dataset derived from a routine TB surveillance system, supporting the relevance of the findings to real-world programmatic settings. The analysis considered multiple outcomes, including treatment success, mortality, and time to treatment initiation, allowing for a broader assessment of the TB care pathway. In addition, the use of established statistical approaches supported risk stratification and the identification of high-risk subgroups using routinely available data. These strengths may enhance the applicability of the findings to TB control programs, although readers should interpret the results within the context of surveillance-based data.

A further strength of the study is the alignment between the analytical methods and the structure of the available surveillance data. By focusing on recorded outcomes, the framework supports practical interpretation for routine programmatic settings while retaining an epidemiologically informed structure for organizing disease-stage concepts.

### Limitations

This study has several important limitations. First, the use of routinely collected surveillance data may be subject to missing data, misclassification, and variability in data quality across reporting sites. Important variables, including socioeconomic status, income, education, migrant status, health care-seeking behavior, timing of symptom onset, and distance to health care facilities, may not have been consistently recorded. Their absence may have introduced residual confounding, particularly in the analysis of treatment delay, as unmeasured social and structural barriers may partly explain the observed associations between rural or remote residence and delayed treatment initiation. The study period also overlapped with the COVID-19 pandemic, which may have affected health care-seeking behavior, TB screening activities, diagnostic access, reporting completeness, and time to treatment initiation [41].

Second, the study was based on registered TB cases in NTIP and may not capture undiagnosed cases, unreported cases, or individuals who did not access care. Readers should therefore interpret the findings as reflecting patterns among registered patients rather than the full burden and heterogeneity of TB in the underlying population. Because NTIP includes diagnosed and registered patients with TB, it cannot directly estimate the population-level effect of increased screening on undiagnosed or unreported cases. Readers should therefore interpret the assumed increase in screening or detection-related indicators in the scenario analysis as a programmatic assumption applied to provincial baseline indicators rather than as an effect estimated from the NTIP cohort itself.

Third, although we used the SEIR framework to guide the conceptual organization of disease stages, the available surveillance data did not directly observe susceptible or exposed populations, and we did not empirically estimate or validate SEIR transition parameters. Accordingly, readers should interpret the framework as SEIR-informed rather than as a fully parameterized transmission model. The observational design also limits causal inference; readers should interpret observed associations as predictive or outcome-specific rather than causal. In addition, combining an epidemiologically informed disease-stage framework with individual-level predictive models requires cautious interpretation; researchers should not directly infer population-level concepts from individual-level associations.

Finally, although we performed internal validation using train-test splitting and cross-validation, we did not conduct external validation using independent datasets. The models may perform differently in settings with varying epidemiological profiles, health care access patterns, population characteristics, reporting completeness, or data quality. The use of multiple predictors and outcome-specific models may also increase the risk of overfitting, particularly in subgroups or lower-frequency outcomes. We based the scenario analysis on predefined assumptions and provincial baseline indicators rather than observed intervention data; therefore, readers should interpret projected changes as illustrative programmatic projections. Future studies should compare performance with simpler baseline models, validate calibration and discrimination in independent datasets, examine sensitivity to scenario assumptions, and evaluate feasibility and implementation in routine TB control.

### Conclusions

TB outcomes in Chiang Mai Province reflected the combined influence of clinical vulnerability and health care access barriers. Mortality was more strongly associated with clinical factors, including HIV co-infection, older age, diabetes, and low BMI. In contrast, treatment delay was more closely related to rural or remote residence and lack of health insurance. These findings suggest that TB control strategies should combine intensified clinical monitoring for high-risk patients with improved access to timely diagnosis and treatment for underserved populations.

An SEIR-informed predictive framework using routine surveillance data may support risk stratification and program planning by identifying high-risk groups and access-related delays in the TB care pathway. The findings support practical actions such as active follow-up for patients with HIV co-infection or other clinical vulnerabilities, strengthened outreach and referral pathways in rural and remote areas, improved linkage to care for uninsured patients, and targeted prioritization of follow-up, outreach, and linkage-to-care activities.

Model-based scenario results suggest that improvements in detection-related indicators and treatment coverage could contribute to better TB outcomes under predefined assumptions. However, readers should interpret these projections cautiously because they are based on modeled scenarios rather than observed intervention effects. Before wider implementation, future studies should externally validate the predictive framework in independent datasets and evaluate it prospectively in routine TB control settings to assess feasibility, calibration, clinical usefulness, and impact on decision-making.

## Supplementary material

10.2196/86495 Checklist 1 STROBE checklist.

## Abbreviations

AUC: area under the receiver operating characteristic curve
HR: hazard ratio
ML: machine learning
NTIP: National Tuberculosis Information Program
SEIR: susceptible-exposed-infectious-recovered
SEIR-D: susceptible-exposed-infectious-recovered-death
TB: tuberculosis
WHO: World Health Organization

## References

[1] (2025). Global tuberculosis report 2025. https://iris.who.int/server/api/core/bitstreams/e97dd6f4-b567-4396-8680-717bac6869a9/content.

[2] Tuberculosis profile: Thailand. World Health Organization.

[3] Chinpong K, Thavornwattana K, Armatrmontree P (2022). Spatiotemporal epidemiology of tuberculosis in Thailand from 2011 to 2020. Biology (Basel).

[4] Side S, Mulbar U, Sidjara S, Sanusi W A SEIR model for transmission of tuberculosis.

[5] Adi YA (2024). An investigation of Susceptible–exposed–infectious–recovered (SEIR) tuberculosis model dynamics with pseudo-recovery and psychological effect. Healthcare Analytics.

[6] Tang N, Yuan M, Chen Z (2023). Machine learning prediction model of tuberculosis incidence based on meteorological factors and air pollutants. Int J Environ Res Public Health.

[7] Bartl L, Zeeb M, Kälin M (2025). Machine learning-based prediction of active tuberculosis in people with HIV using clinical data. Clin Infect Dis.

[8] Verboven L, Calders T, Callens S (2022). A treatment recommender clinical decision support system for personalized medicine: method development and proof-of-concept for drug resistant tuberculosis. BMC Med Inform Decis Mak.

[9] Wang M, Lee C, Wei Z, Ji H, Yang Y, Yang C (2023). Clinical assistant decision-making model of tuberculosis based on electronic health records. BioData Min.

[10] Hethcote HW (2000). The mathematics of infectious diseases. SIAM Rev.

[11] Jittimanee S, Vorasingha J, Mad-asin W, Nateniyom S, Rienthong S, Varma JK (2009). Tuberculosis in Thailand: epidemiology and program performance, 2001-2005. Int J Infect Dis.

[12] Division of Tuberculosis, Department of Disease Control (2022). The report on the implementation of the National Tuberculosis Control Program 2016–2020.

[13] Division of Tuberculosis, Department of Disease Control (2022). Report of the 6th Tuberculosis Joint International Monitoring Mission Thailand: 29 May to 6 June 2022.

[14] Rubin DB (1987). Multiple Imputation for Nonresponse in Surveys.

[15] van Buuren S (2018). Flexible Imputation of Missing Data.

[16] Azur MJ, Stuart EA, Frangakis C, Leaf PJ (2011). Multiple imputation by chained equations: what is it and how does it work?. Int J Methods Psych Res.

[17] Hosmer DW, Lemeshow S (2000). Applied Logistic Regression.

[18] Steyerberg EW (2009). Clinical Prediction Models: A Practical Approach to Development, Validation, and Updating.

[19] Cox DR (1972). Regression models and life-tables. J R Stat Soc Series B Stat Methodol.

[20] Kleinbaum DG, Klein M (2012). Survival Analysis: A Self-Learning Text.

[21] Keeling MJ, Rohani P (2008). Modeling Infectious Diseases in Humans and Animals.

[22] Hanley JA, McNeil BJ (1982). The meaning and use of the area under a receiver operating characteristic (ROC) curve. Radiology.

[23] Harrell FE, Califf RM, Pryor DB, Lee KL, Rosati RA (1982). Evaluating the yield of medical tests. JAMA.

[24] Dowdy DW, Dye C, Cohen T (2013). Data needs for evidence-based decisions: a tuberculosis modeler’s “wish list”. Int J Tuberc Lung Dis.

[25] Lelisho ME, Wotale TW, Tareke SA (2022). Survival rate and predictors of mortality among TB/HIV co-infected adult patients: retrospective cohort study. Sci Rep.

[26] Xie Y, Han J, Yu W, Wu J, Li X, Chen H (2020). Survival analysis of risk factors for mortality in a cohort of patients with tuberculosis. Can Respir J.

[27] Bello S, Afolabi RF, Ajayi DT (2019). Empirical evidence of delays in diagnosis and treatment of pulmonary tuberculosis: systematic review and meta-regression analysis. BMC Public Health.

[28] Arriaga MB, Araújo-Pereira M, Barreto-Duarte B (2022). The effect of diabetes and prediabetes on antituberculosis treatment outcomes: a multicenter prospective cohort study. J Infect Dis.

[29] Bezerra AL, da Silva Rezende Moreira A, Isidoro-Gonçalves L (2022). Clinical, laboratory, and radiographic aspects of patients with pulmonary tuberculosis and dysglycemia and tuberculosis treatment outcomes. J Bras Pneumol.

[30] Burke RM, Nliwasa M, Feasey HRA (2021). Community-based active case-finding interventions for tuberculosis: a systematic review. Lancet Public Health.

[31] Bohlbro AS, Hvingelby VS, Rudolf F, Wejse C, Patsche CB (2021). Active case-finding of tuberculosis in general populations and at-risk groups: a systematic review and meta-analysis. Eur Respir J.

[32] Rashidi HH, Dang LT, Albahra S, Ravindran R, Khan IH (2021). Automated machine learning for endemic active tuberculosis prediction from multiplex serological data. Sci Rep.

[33] Wang Z, Guo Z, Wang W (2025). Prediction of tuberculosis treatment outcomes using biochemical makers with machine learning. BMC Infect Dis.

[34] K B HM, Jose SA, Jirawattanapanit A, Mathew K (2025). A comprehensive study on tuberculosis prediction models: integrating machine learning into epidemiological analysis. J Theor Biol.

[35] Biswas MHA, Paiva LT, de Pinho MDR (2014). A SEIR model for control of infectious diseases with constraints. Math Biosci Eng.

[36] Ucakan Y, Gulen S, Koklu K (2021). Analysing of tuberculosis in Turkey through SIR, SEIR and BSEIR mathematical models. Math Comput Model Dyn Syst.

[37] Ghosh A, Das P, Das SK, Das P, Upadhyay RK (2025). Forecasting tuberculosis through mechanistic learning of transmission dynamics: insights from a case study in India. Comput Biol Med.

[38] Shaweno D, Karmakar M, Alene KA (2018). Methods used in the spatial analysis of tuberculosis epidemiology: a systematic review. BMC Med.

[39] Faccin M, Geenen C, Happaerts M, Ombelet S, Migambi P, André E (2025). Analyzing satellite imagery to target tuberculosis control interventions in densely urbanized areas of Kigali, Rwanda: cross-sectional pilot study. JMIR Public Health Surveill.

[40] Lee Y, Raviglione MC, Flahault A (2020). Use of digital technology to enhance tuberculosis control: scoping review. J Med Internet Res.

[41] McQuaid CF, Vassall A, Cohen T, Fiekert K, White RG (2021). The impact of COVID-19 on TB: a review of the data. Int J Tuberc Lung Dis.

